# Geochemical characteristics and provenance of the detrital sediments in the junction area of Yinggehai and Qiongdongnan basins, South China Sea

**DOI:** 10.1038/s41598-023-28778-8

**Published:** 2023-01-30

**Authors:** Fulin Meng, Huajun Gan, Hua Wang, Si Chen, Yin Gong, Qiyang Zhang

**Affiliations:** 1 Key Laboratory of Tectonics and Petroleum Resources, China University of Geosciences, Ministry of Education, Wuhan, 430074 China; 2 School of Earth Resources, China University of Geosciences, Wuhan, 430074 China; 3 The Seventh Geological Brigade of Hubei Geological Bureau, Yichang, 443100 China

**Keywords:** Geochemistry, Geology, Sedimentology

## Abstract

Together, the Yinggehai and Qiongdongnan basins have received a large amount of terrigenous sediments, but the provenance evolution of Cenozoic sediments in the two basins remains disputable. Combined with previous studies in the Yinggehai and Qiongdongnan basins, the elemental geochemistry of Oligocene to Pliocene sediment samples in the junction area of the two basins were analyzed to explore the tectonic implications, parent rock characteristics, and provenance evolution of the two basins during the Cenozoic. The results reveal that all the sediment samples were derived from continental island arc to passive continental margin settings. The light REE enrichment and stable content of heavy REE with large negative Eu anomalies indicate that they were probably derived from Hainan Island. The reconstructed provenance evolution model showed that the Red River Source (RRS) provided sedimentary materials for the Central Depression of Yinggehai Basin from the Oligocene to the Pliocene, and Hainan Island Source (HIS) was also one of the sources for sediments deposited in the Central Depression of Yinggehai Basin during the Miocene. However, most of the sediments preserved in the Yingdong Slope and Qiongdongnan Basin were derived from the HIS from the Oligocene to the Pliocene, and sediments deposited in the Yingdong Slope were also derived from the RRS during the Miocene. Furthermore, the junction area of the two basins had a mixed source of the RRS and HIS during the Cenozoic.

## Introduction

Large oil and gas reservoirs have been discovered in the Yinggehai and Qiongdongnan basins recently^1–6^. The Yinggehai and Qiongdongnan basins are located in northwestern part of the South China Sea (SCS), which received a large amount of sediments from the Red River to the northwest, the Hainan Island to the east and north, and Central Vietnam to the west during the Cenozoic^7–10^. Many records of the tectonic evolution, denudation characteristics, and climate change of the surrounding potential source areas have been achieved from Cenozoic sediments^11–14^.

Provenance analysis plays a critical role in determining source areas, sediment transport route, and basin evolution^3,12,14,15^. During weathering, transportation, sedimentation, diagenesis, and low-grade metamorphism, the chemical properties of rare earth elements (REE) are rarely changed, and are truly immobile, with low solubilities and high stabilities^16–18^. Stable REE are usually used as important tracers when studying sedimentary source evolution. The most fundamental reason for this is that REE exhibit a very strong inheritance from their parent rocks^19,20^. Previous provenance studies in the Yinggehai and Qiongdongnan basins were mainly based on heavy mineral, seismic, and mineralogical methods^5,7,15^, and both elemental geochemistry and detrital zircon ages have been adopted for provenance studies in the Yinggehai and Qiongdongnan basins during recent years^9,13,21,22^. Elemental geochemistry and detrital zircon ages from the Yinggehai Basin have indicated a continuous record of sedimentary provenance from the Red River since the Oligocene^12,21–24^. Furthermore, rivers originating from Hainan Island provided a lot of sediments in the Qiongdongnan Basin, and sediments provided by the Red River entered the Qiongdongnan Basin as early as the late Oligocene^2,14^.

**Figure 1:**
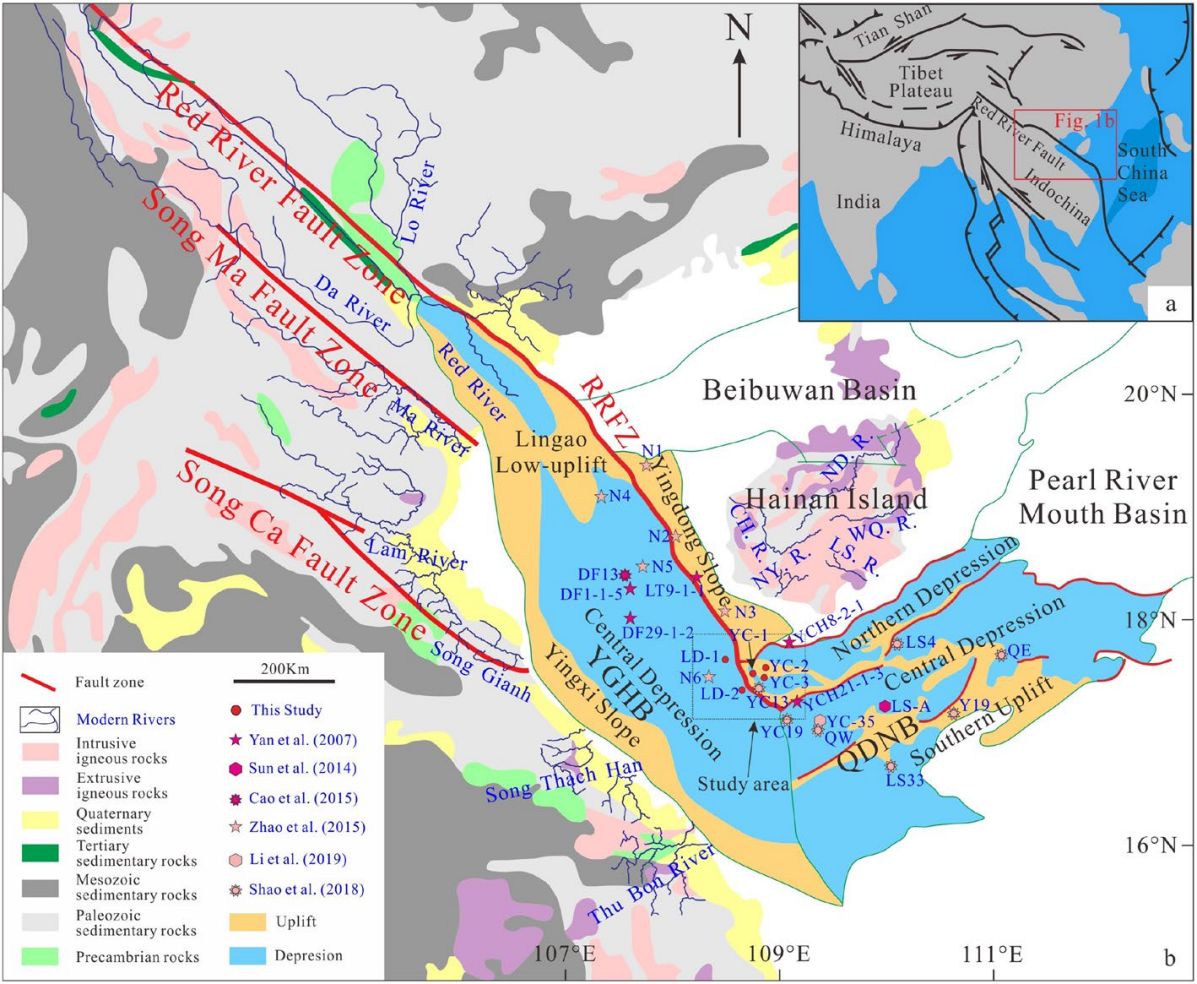
Map of tectonics and geology of Southeast Asia (modified from^7^) show the location of Yinggehai and Qiongdongnan basins (**a**). Tectonic zones of the Yinggehai and Qiongdongnan basins (modified from^14^) and the simplified geological map (modified from^8^) of the potential source areas showing the lithological distribution along river cources (**b**). Major fault zones and major drainage system modified from^22^. YGHB-Yinggehai Basin, QDNB-Qiongdongnan Basin, ND. R.-Nandu River, CH. R.-Chuanghua River, NY. R.-Ningyuan River, LS. R.-Lingshui River, WQ. R.-Wanquan River.

Previous studies of the Yinggehai and Qiongdongnan basins have focused on individual tectonic settings and stratigraphic units, however systematic studies of the sedimentary provenance in the junction area of two basins have been ignored. Hainan Island Source (HIS), Red River Source (RRS), and Central Vietnam Source (CVS) are three potential sources of detrital sediment for the Yinggehai and Qiongdongnan basins during the Cenozoic, and our study area is located at the junction of the two basins (Fig. 1). However, the study area is the only passageway for the RRS to enter the Qiongdongnan Basin, and it is an ideal area to detect whether the detrital sediments from the RRS have been transported through the junction of the two basins. In this study, sandstone core samples were collected from five wells in different formations of Oligocene to Pliocene age for geochemical analysis. The aims of this paper are to explore the tectonic implications, analyse the parent rock characteristics and accurately trace the source of detrital sediments in this area. We combine these new data with results of previous studies to further explore the provenance evolution of the Yinggehai and Qiongdongnan basins during the Cenozoic.

## Geological setting

### Geological background of Yinggehai and Qiongdongnan basins

The NNW-SSE trending Yinggehai Basin, which is located on the continental shelf of SCS, is surrounded by the Red River and the Beibuwan Basin to the north, Hainan Island to the east, and bordered on the west by Central Vietnam and southeast by Qiongdongnan Basin, respectively (Fig. 1). The Yingxi Slope, Yingdong Slope, and Lingao Low-uplift are the western, eastern, and northern parts of the basin, respectively, and the main deposited area of Yinggehai Basin is the Central Depression (Fig. 1). A large amount of Cenozoic sediment was deposited in the Yinggehai Basin, and strata thickness in the center of the basin is up to 17 km^7,25,26^.

The Qiongdongnan Basin is a Cenozoic gas-bearing marine basin, and its long axis trends from southwest to northeast. It is bounded by Hainan Island in the north, Pearl River Mouth Basin in the west, and SCS in the south^2,7,8^ (Fig. 1). The Qiongdongnan Basin can be divided into three tectonic zones: the Northern and Central Depressions are close to Hainan Island and distributed in a NE-SW direction. The Southern Uplift is high in the middle and low in the northeast and southwest^3,14,22,27^ (Fig. 1).

**Figure 2:**
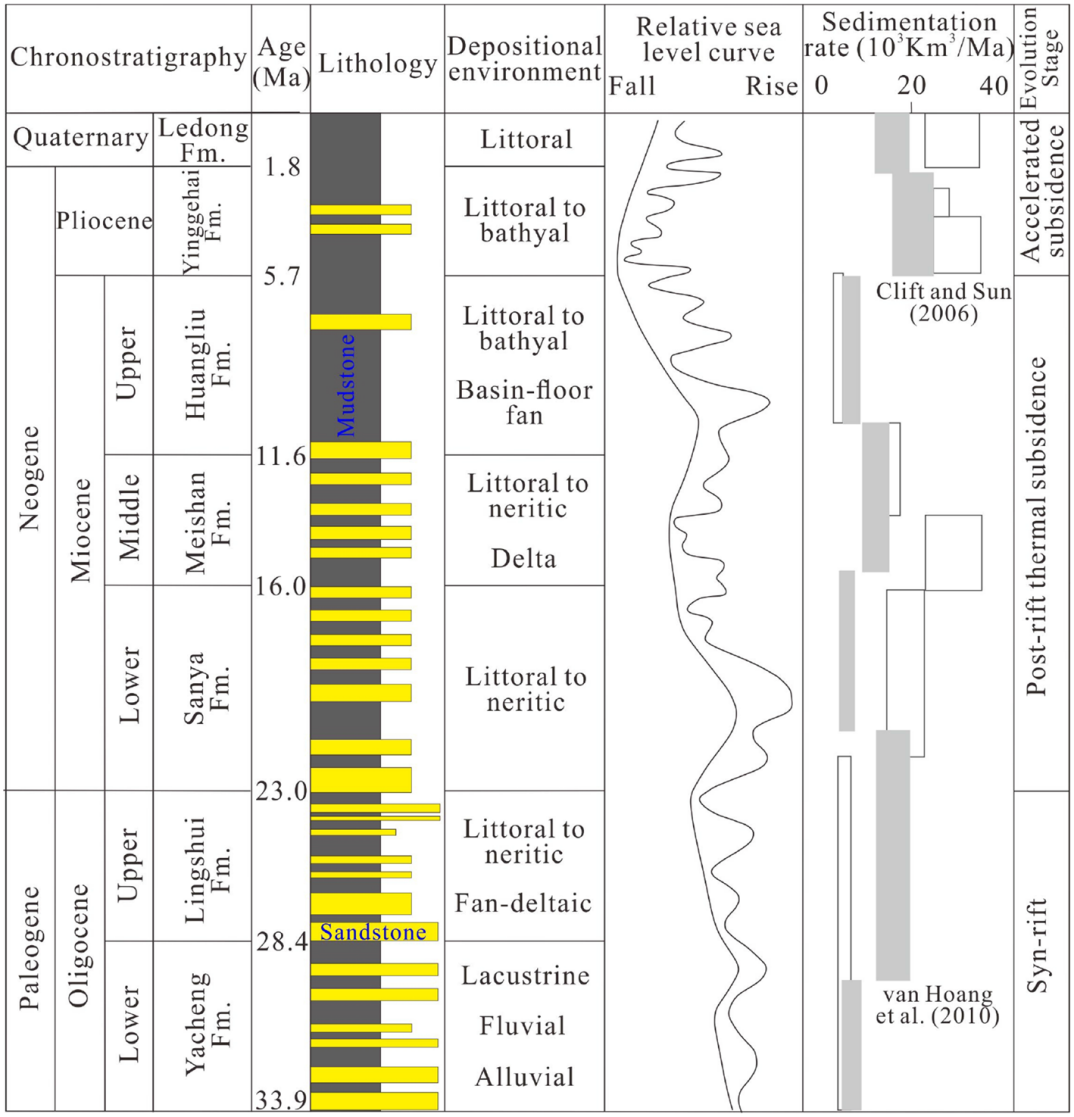
Cenozoic stratigraphic table of the Yinggehai and Qiongdongnan basins with lithostratigraphy, relative sea level curve, sedimentation rate, and regional tectonic events^8,14,26^.

Most previous studies suggest that the basins have experienced an early syn-rifting stage and later post-rifting stage (Fig. 2)^7,25,28^. During the syn-rifting stage, lacustrine, fluvial, and alluvial deposits were formed in the Eocene, while the later Oligocene strata are characterized by coastal and shallow marine sediments^13,25,29,30^. The post-rifting stage has been divided into steady thermal subsidence and rapid subsidence sub-stages by the abrupt change of the basins’ subsidence rates in the late Miocene, which correspond to the Miocene and Pliocene to Quaternary, respectively (Fig. 2)^7,26,30,31^. During the steady thermal subsidence sub-stage, the basins underwent thermal subsidence, characterized by attenuated fault activity. Moreover, tectonic inversion widely occurred in the northern part of the Yinggehai Basin due to the regional stress pattern switching from transtensional to transpressional^7,26,32^. Sea level fluctuated greatly during Miocene, and sedimentation was characterized by littoral and deltaic facies to neritic facies^7,8,30^. During the rapid subsidence sub-stage, the two basins experienced the similar subsidence rates, and the sedimentation was characterized by neritic-bathyal facies^8,29,33,34^.

### Provenance hypotheses

Previous studies have suggested three dominant source areas, including RRS, HIS, and CVS (Fig. 1). The sediment mainly originated from the mafic–ultramafic gneiss, schists, ophiolites, intermediate-mafic volcanic rocks, and sedimentary rocks in RRS^8,11,24,35^, and the Red River system delivered a large volume of sediment to the Yinggehai or Qiongdongnan basins. However, the Cenozoic capture of the upstream Red River has been debated for years^36–40^. Hainan Island is high in the central mountains and low in the peripheral plains, and many rivers originate from the center of Hainan Island and flow into the surrounding basins. The Nandu River flows into the Beibuwan Basin, the Chuanghua River and Ningyuan River flow into the Yingdong Slope in the Yinggehai Basin, and the Lingshui River and Wanquan River flow into the Northern Depression in the Qiongdongnan Basin (Fig. 1), resulting in a spokewise provenance system (HIS). Except for the limited modern basalts in the northern area, almost all parent rocks from the HIS are intermediate-felsic intrusions^8,12^ (Fig. 1). Hainan Island underwent a rapid cooling phase from late Eocene to Oligocene^23^. From north to south, several rivers (e.g., the Ma, Lam, Song Gianh, Song Thach Han, and Thubon rivers) originate from Central Vietnam and flow into the Yingxi Slope of Yinggehai Basin. The parent rocks in the CVS are Paleozoic–Mesozoic sedimentary rocks and a limited amount of rhyolite and mafic rocks^8,21^ (Fig. 1). However, it is suggested that the Central Vietnam source supplied only 17–21% of the total sediments now preserved in the Yinggehai Basin based on apatite fission track data and ^10^Be data^41^.

## Samples and methods

### Samples

A total of 39 samples were collected from five wells to analyze the geochemical characteristics of Oligocene to Pliocene sediments in the study area. The detailed information on the samples is listed in Table 1. All the samples are sandstone from the drill cores.

**Table 1:** Stratigraphic positions of the samples analyzed in this study.

Well	Number of samples	Age	Formation	Lithology	Material
LD-1	2 (1, 2)*	Pliocene	Yinggehai	Sandstone	Core
LD-2	4 (3–6)	Late Miocene	Huangliu	Sandstone	Core
YC-1	4 (7–10)	Late Miocene	Huangliu	Sandstone	Core
YC-2	4 (11–14)	Late Miocene	Huangliu	Sandstone	Core
YC-3	8 (15–22)	Middle Miocene	Meishan	Sandstone	Core
YC-3	6 (23–8)	Early Miocene	Sanya	Sandstone	Core
YC-3	9 (29–37)	Late Oligocene	Lingshui	Sandstone	Core
YC-3	2 (38, 39)	Early Oligocene	Yacheng	Sandstone	Core

*Total (Sample No).

### Methods

Before major element geochemical analysis, we mixed 0.6 g of each sample with flux at a ratio of 1:10, using a flux was of Li_2_B_4_O_7_, LiBO_2_, and LiF in a ratio of 9:2:1. NH_4_NO_3_ was used as an oxidant to fully melt the sample during digestion. This mixture was heated into a glass bead, and X-ray fluorescence spectroscopy (ZSX Primus II, RIGAKU, Japan) was conducted on the fused glass beads to determine the major elemental concentrations. The melting temperature and time were 1050 °C and 15 min, respectively. The concentrations of the major elements in the whole-rock samples were determined at a voltage of 50 kV and a current of 60 mA. The theoretical α coefficient method was used to correct the data, and the relative standard deviation of the corrected data is less than 2%. This analysis was carried out at the Wuhan Sample Solution Analytical Technology Co., Ltd., Wuhan, China.

An Agilent 7700e inductively coupled plasma mass spectrometer (ICP-MS) was used to analyze the trace element and REE concentrations of the whole-rock samples at the Wuhan Sample Solution Analytical Technology Co., Ltd., Wuhan, China. We used a mixture of HNO_3_ and HF for pressurized acid digestion of the samples. The minimum detection limit of the equipment is < 1 ppb, and the errors of the trace element and REE data obtained are within ± 6%. The trace element and REE analysis was conducted according to the detailed treatment process and analytical procedures described by reference^42^.

## Results

Supplementary Table 1 presents the geochemical data obtained in this study, including major elemental data for 16 samples, trace elemental data for 39 samples, and REE data for 39 samples.

### Major elements

**Figure 3:**
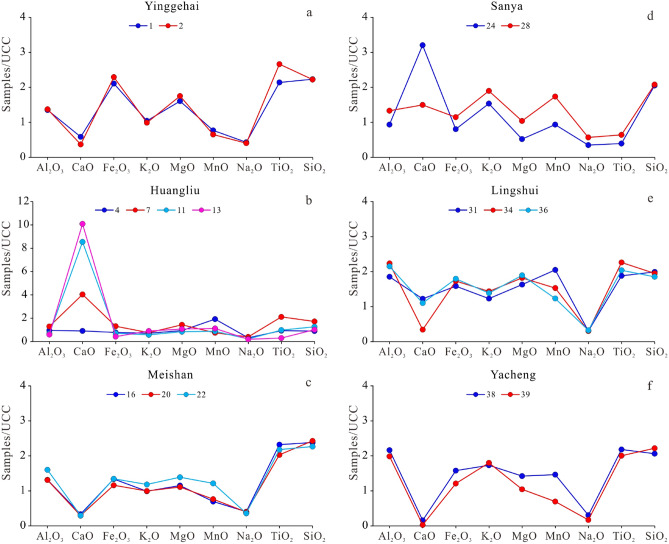
Normalized major elements of the upper continental crust^43^ of Cenozoic sediment samples obtained from five wells in the junction area of Yinggehai and Qiongdongnan basins.

**Figure 4:**
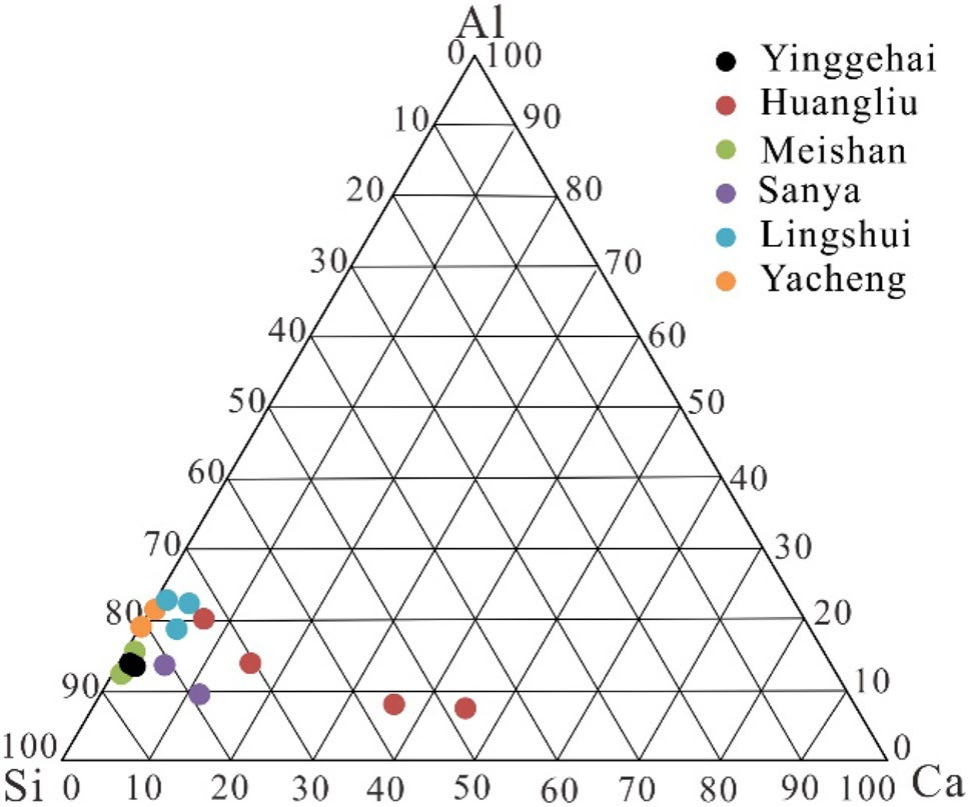
Ca–Si–Al ternary diagram of Cenozoic sediment samples obtained from five wells in the junction area of Yinggehai and Qiongdongnan basins.

The variation in the major elemental concentrations of samples is illustrated by the plot normalized to the Upper Continental Crust (UCC) (Fig. 3), and there are different distribution patterns among different formations. Compared to the Sanya and Huangliu formations (Fig. 3d and b), the UCC-normalized elemental distributions of the Yacheng, Lingshui, Meishan, and Yinggehai formations exhibit noticeable Ca and Na depletions (Fig. 3f,e,c, and a, respectively). Compared with the other formations, the UCC-normalized elemental distribution of the Huangliu formation exhibits obvious Ca enrichment (Fig. 3b). The Yinggehai formation exhibits significant Ca, K, and Na depletion and significant Fe, Mg, and Ti enrichment (Fig. 3a). The UCC-normalized major elemental distribution of the Sanya formation exhibits Ca and Si enrichment and Na and Ti depletion (Fig. 3d). Except for the Huangliu formation, most of the data for the Cenozoic samples are plotted in or close to the Si apex, suggesting that the clastic silicates are the major component of the Cenozoic sediments from the study area (Fig. 4).

### Trace elements

**Figure 5:**
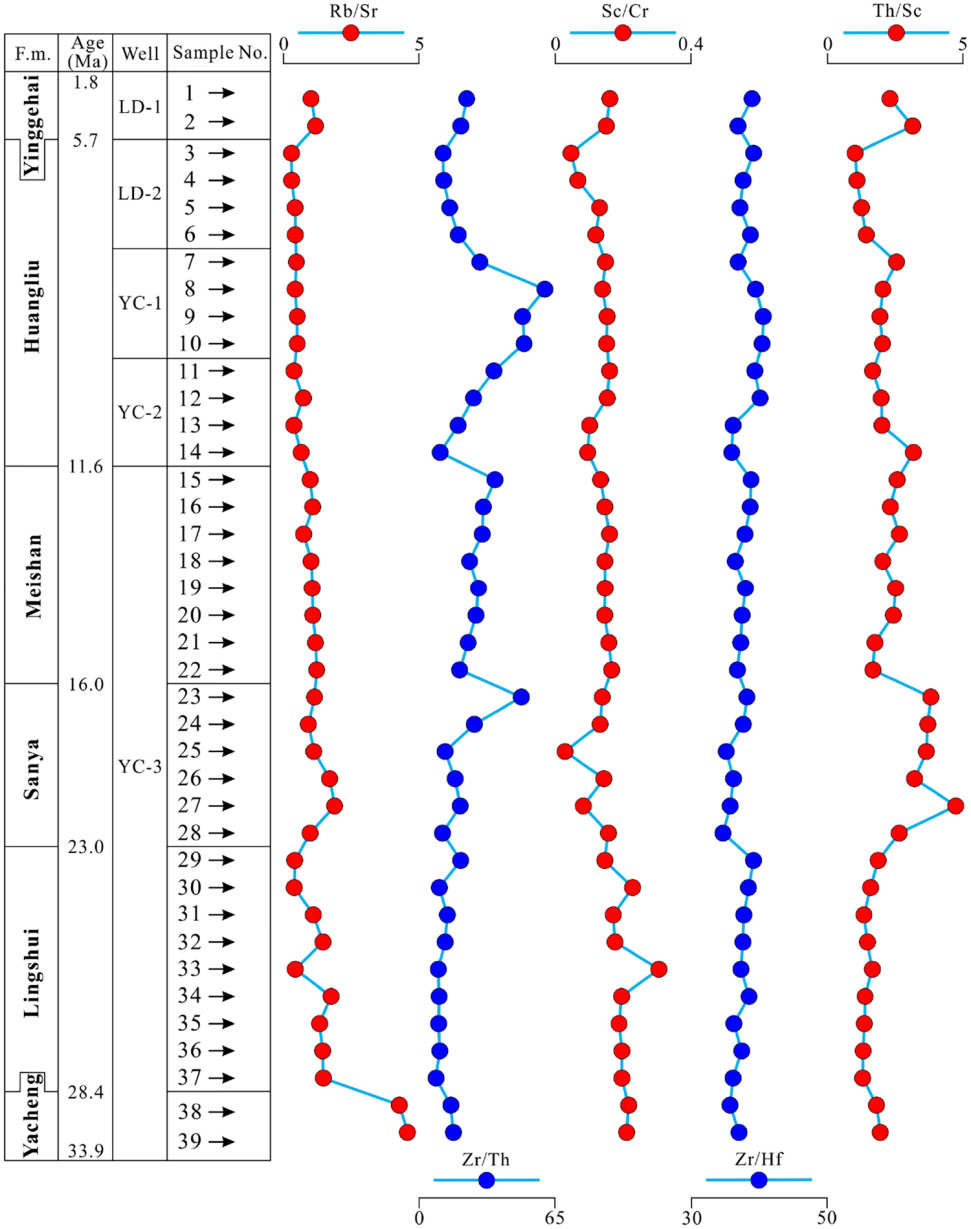
Comparative variations in the trace elemental ratios from Wells in the junction area of Yinggehai and Qiongdongnan basins, F.M.-Formation.

The trace elements of Th, Zr, and Hf are relatively stable and exhibit a very strong inheritance from their parent rocks, so they are often used for identifying tectonic environments^44,45^. In general, Sc content and Sc/Cr, Ti/Zr, and Ba/Rb ratio increase, and Rb and Th content and Th/Sc and Rb/Sr ratio decrease systematically from the passive marginal environment to the active continental marginal environment, then to continental island arc environment, and then to continental island arc environment^46,47^. Five trace elemental ratios (Rb/Sr, Zr/Th, Sc/Cr, Zr/Hf and Th/Sc) of Cenozoic samples show overall stability, with slight local changes from the Oligocene to Pliocene (Fig. 5). The Rb/Sr, Sc/Cr, and Zr/Hf ratios exhibit less variation, while the Zr/Th and Th/Sc ratios fluctuate with a high frequency in the Sanya and Huangliu formations. Furthermore, the average Rb/Sr ratio of the Yacheng formation is 4.39, which is much higher than those of the upper formations (Fig. 5). In the Huangliu formation, the Zr/Th ratio ranges from 10.12 to 60.12, with an average of 28.02, higher than those of the other formations. The Th/Sc ratio of samples varies from 1.03 to 4.73, which is similar to sandstones whose parent rocks are felsic rocks.

### Rare earth elements

**Figure 6:**
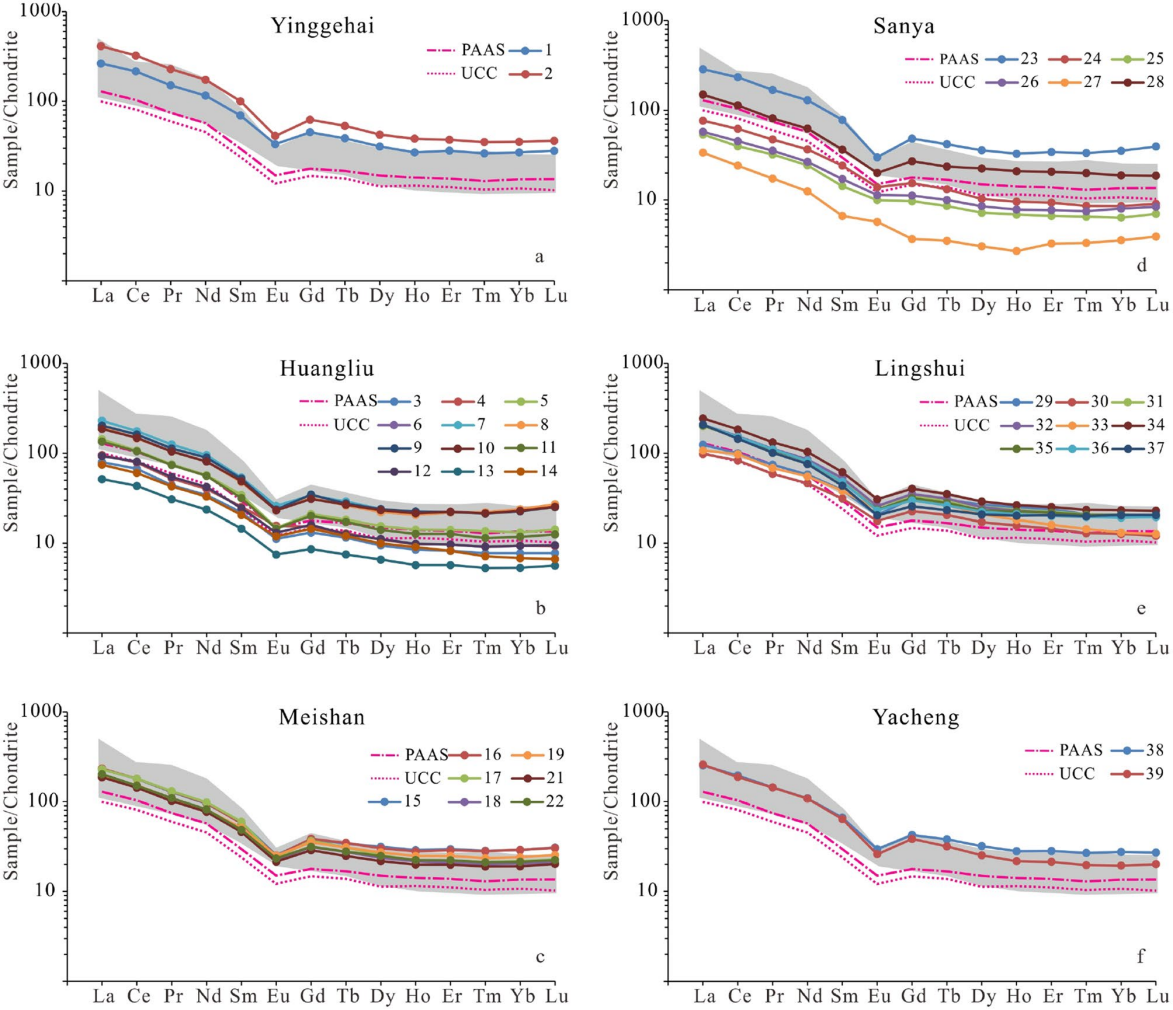
Chondrite^50^ normalized REE concentrations in Cenozoic sediments from the junction area of Yinggehai and Qiongdongnan basins. Grey area indicates sediments likely from Hainan Island source^51^.

Table 2 shows the statistical results of the REE contents of the Cenozoic sediments from the five wells in the study area. The average REE concentrations in all samples are higher than those of Post-Archean Australian shale (PAAS) (184.77 × 10^−6^), but the average REE concentrations of samples in the Sanya formation from well YC-3 and the Huangliu formation from wells LD-2 and YC-1 are significantly lower than those of UCC (146.37 × 10^–6^). The samples from the five wells are significantly enriched in light REE (LREE) relative to heavy REE (HREE), which is illustrated by the *LREE/HREE* ratios of 7.95–9.44. Furthermore, the obvious fractionation between the LREE and HREE can also be seen from the average (La/Yb)_N_ ratios, ranging from 8.34 to 11.28. However, the average (La/Sm)_N_ values range from 3.8 to 4.02, indicating marked fractionation of the LREE (Table 2). The chondrite-normalized REE distribution patterns of the samples are nearly parallel, with negative Eu anomalies similar to UCC and PAAS (Fig. 6). Based on the average δEu values from 0.53 to 0.76, the Cenozoic sediments in the study area were significantly influenced by intermediate-felsic volcanic sources (Table 2). Generally, the δCe value of sedimentary rocks, which parent rocks derived from terrigenous non-carbonate sediments, ranges from 0.8 to 1.20^48^. The average δCe values of the Cenozoic sediments in the study area range from 0.96 to 1.05, with no significant Ce anomalies, suggesting that these sediments have average δCe values similar to those of sediments deposited in a continental environment (from 0.8 to 1.20) during the Cenozoic^48^. In addition to distribution patterns similar to UCC and PAAS, the Cenozoic sediment samples also have large negative Eu anomalies and no significant Ce anomalies.

**Table 2:** Major rare earth elements (REE) characteristics of the Cenozoic sediments from the junction area of Yinggehai and Qiongdongnan basins.

Formation	Well	∑REE (ppm)	$\frac{LREE}{HREE}$	(La/Yb)_N_	(La/Sm)_N_	(Gd/Yb)_N_	δEu	δCe
Yinggehai	LD-1	381.04	9.44	10.71	3.95	1.72	0.54	1.03
Huangliu	LD-2	125.81	9.26	10.59	3.96	1.64	0.59	1.02
Huangliu	YC-1	229.24	8.67	8.83	4.02	1.42	0.58	1.00
Huangliu	YC-2	102.27	9.09	10.46	3.8	1.79	0.63	1.05
Meishan	YC-3	233.56	8.31	8.98	3.94	1.46	0.55	0.99
Sanya	YC-3	127.28	8.07	8.34	3.85	1.43	0.76	0.98
Lingshui	YC-3	199.83	7.95	9.35	3.83	1.68	0.61	0.99
Yacheng	YC-3	281.82	9.31	11.28	3.94	1.76	0.53	0.96
Oceanic island arc^a^		58 ± 10	3.8 ± 0.9	2.8 ± 0.9	–	–	1.04 ± 0.11	–
Continental island arc^a^		146 ± 20	7.7 ± 1.7	7.5 ± 2.5	–	–	0.79 ± 0.13	–
Active continental margin^a^		186	9.10	8.5	–	–	0.6	–
Passive continental margin^a^		210	8.50	10.8	–	–	0.56	–

*∑REE is the total REE content (ppm); $\frac{LREE}{{HREE}}$ is the light to heavy rare earth elemental ratio; δEu = $\frac{Eu}{{Eu*}}$  = $\frac{2*EuN}{{SmN + GdN}}$; δCe = $\frac{Ce}{{Ce*}}$  = $\frac{2*CeN}{{LaN + PrN}}$; a denotes data from^49^.

## Discussion

### Tectonic implications

**Figure 7:**
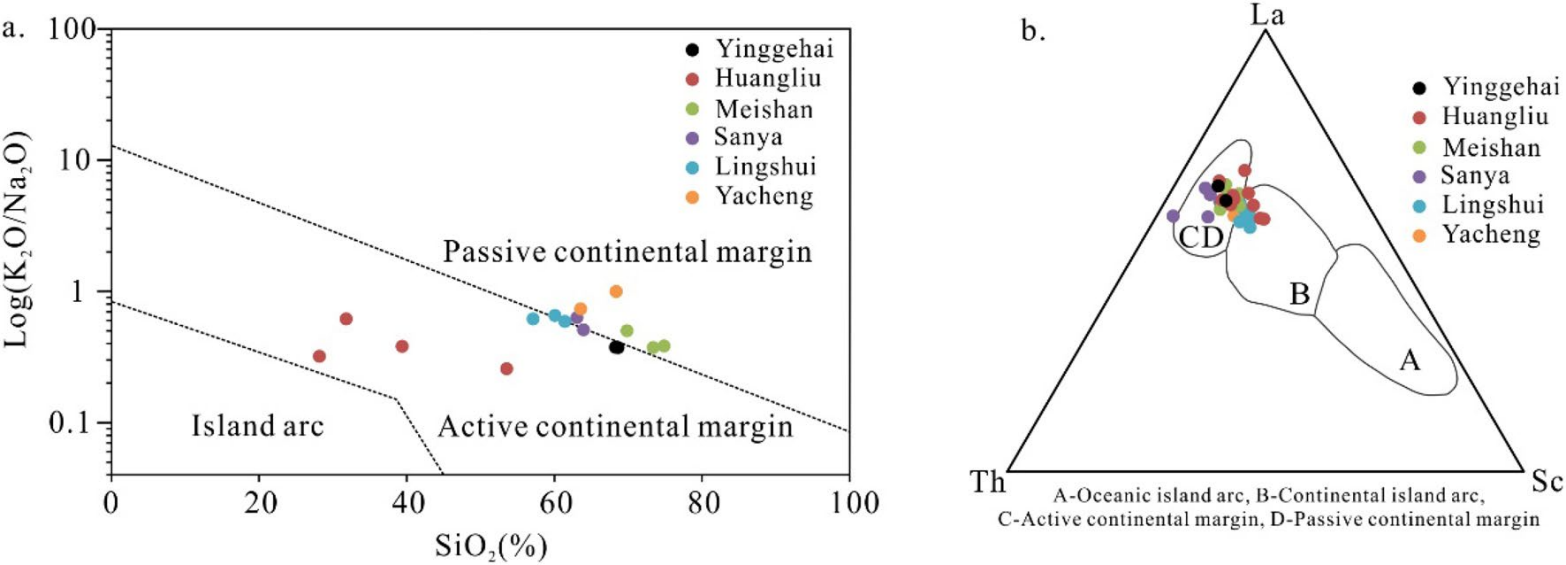
(**a**) Plot of SiO_2_ vs K_2_O/Na_2_O and (**b**) ternary plot of Th-La-Sc to discriminate tectonic setting^49,53^.

Tectonically, the Yinggehai and Qiongdongnan basins are located at the intersection of the Indochina Block, the Pacific Plate, and the southern margin of the South China Block^31,35,52^. Considerable information about the regional history has likely been recorded in the Cenozoic sediments from the Yinggehai and Qiongdongnan basins. Geochemical data could better interpret the regional tectonic background^45,53^. The samples of Cenozoic sediments from the study area are significantly enriched in LREE (Table 2) and show large negative Eu anomalies (Fig. 6), which have similar REE enrichment and distributed characteristics to andesite developed in continental island arc and passive continental margin environment^49^. According to the plot of SiO_2_ versus K_2_O/Na_2_O, except for the Huangliu formation samples, all the samples plot in the passive continental margin field (Fig. 7a). Furthermore, most Lingshui and Huangliu formation samples plot in the continental island arc field, while the Yacheng, Sanya, Meishan, and Yinggehai formations’ samples plot in the active and passive continental margin fields (Fig. 7b). The Cenozoic sediments in the study area were derived from the continental island arc and passive continental margin setting.

### Parent rock characteristics

The distribution patterns of the major elemental chemistry of the samples in different formations are generally similar. The most obvious characteristic is that CaO and Na_2_O relative abundance decline significantly through time, while the Sanya and Huangliu formations are enriched in CaO relative to those from other formations (Fig. 3). The Ca and Na are degraded quickly during the process of weathering and transport on account of smectites, chlorites, and other clays which house Ca and Na are susceptible to weathering^54,55^, which may cause the relative abundance of CaO and Na_2_O decrease significantly. Due to the massive sea level fall during deposition of the Sanya and Huangliu formation, the calcium-rich minerals in the sediment source increased significantly^30,56^. As demonstrated in the previous studies, the REE of sediments from the HIS is characterized by enrichment of LREE and stable content of HREE with negative Eu anomalies^51^, while the sediments provided by the RRS exhibit positive Eu anomalies^24^, or unstable content of HREE with negative Ce and Eu anomalies^57^. The chondrite-normalized REE distribution patterns from our study are more consistent with those from the HIS, which indicate that the sediments in the study area are originated from HIS (Fig. 6).

**Figure 8:**
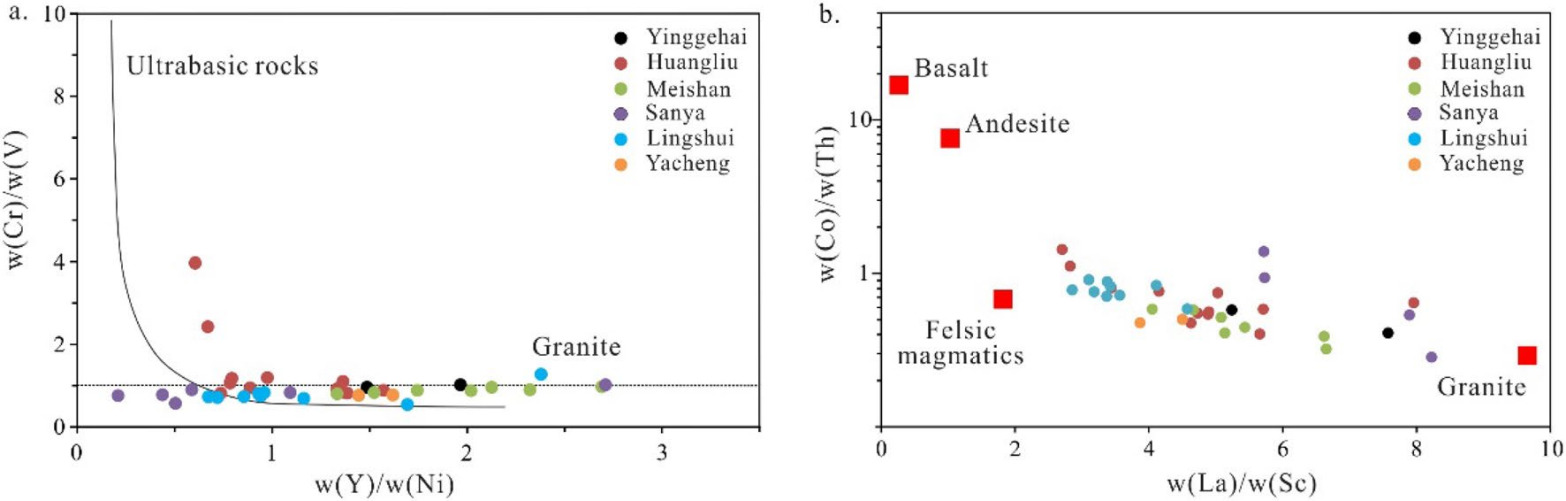
(**a**) Plot of Cr/V vs Y/Ni and (**b**) plot of Co/Th vs La/Sc.

**Figure 9:**
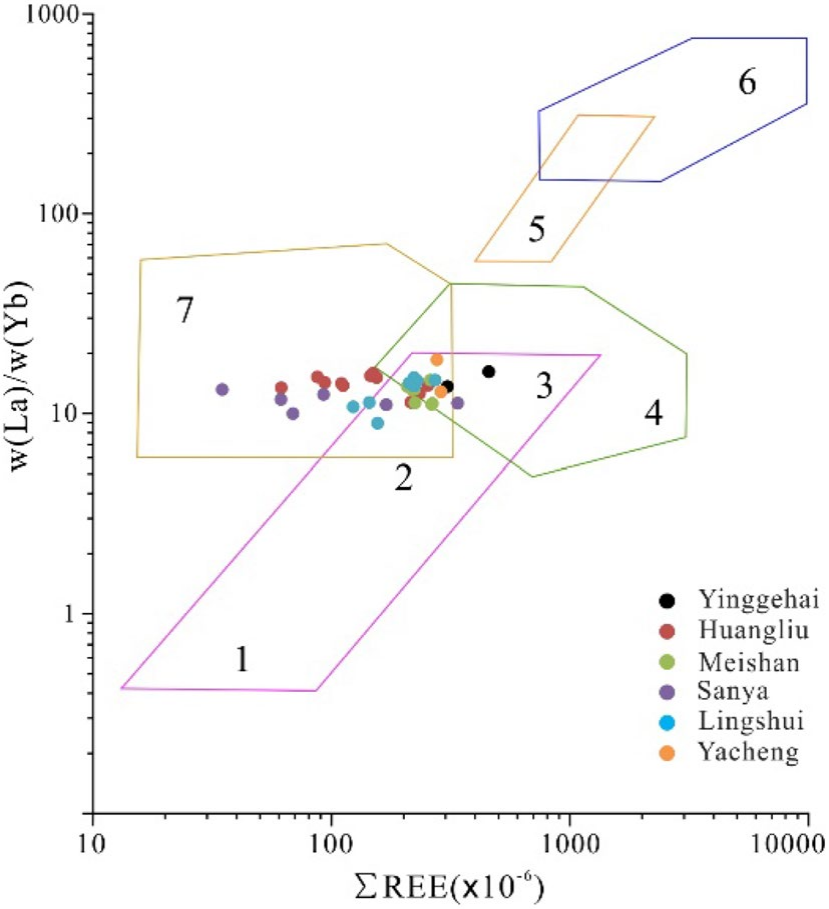
Parent rock discrimination from La/Yb vs ∑REE for Cenozoic sediments from the junction area of Yinggehai and Qiongdongnan basins^58^. 1: oceanic tholeiitic basalt, 2: continental tholeiitic basalt, 3: alkali basalt, 4: granite, 5: kimberlite, 6: carbonate rocks, and 7: sedimentary rock and calcareous mudstone.

When analyzing the sources of sediments, it is more effective to use the ratios of immobile elements to mobile elements than single elemental concentrations due to the geochemical stability of high field strength elements during transport and deposition^46–48^. Moreover, sediments derived from mafic and felsic parent rocks have significantly different trace element and REE contents^46,47^. Therefore, the parent rock information of sedimentary rocks can be identified by the ratios of La/Sc, Co/Th, Cr/V, and Y/Ni. Based on the cross plot of Cr/V–Y/Ni (Fig. 8a) and Co/Th-La/Sc (Fig. 8b), most of the Cenozoic sediments from the study area are plotted in or close to the felsic volcanic and granite fields. To furtherly investigate the nature of the parent rocks, the samples were plotted in the La/Yb-∑REE diagram (Fig. 9). It is shown that most of the samples are plotted at the intersection of the basalt, granite, and sedimentary rock fields, but samples of Sanya and Huangliu formations are plotted closer to the sedimentary rock field. Overall, the parent rocks of Cenozoic sandstone from the study area seem to be unique, indicating that these sediments are derived from Hainan Island, which contains igneous and ancient sedimentary rocks.

### Provenance evolution

Based on seismic profiles and petrographic data of sandstones, previous studies have shown that the RRS, HIS, and CVS provided large amounts of sediment to the Yinggehai Basin^7,25,32,59^. Moreover, recently published detrital zircon U–Pb dating provenance studies have revealed that HIS was likely the major source of sediments in the Yingdong slope, but the Central Depression of Yinggehai Basin received a large amount of terrigenous sediment from both RRS and HIS during the Cenozoic^8,9,12^. Progradational clinoforms were identified on seismic profiles along the margin of Hainan Island in previous studies, suggesting that Hainan Island also provided sediments to the Qiongdongnan Basin^25,28,29^. Furthermore, the age distribution of Lingshui Formation detrital zircons in the Qiongdongnan Basin shows two major peaks at ca. 247 Ma and 441 Ma, and accompanied by nine subordinate age peaks from 35 to 1905 Ma (consistent with the zircon age distribution of RRS), which indicates that sediments from RRS were deposited in the western part of Qiongdongnan Basin as early as late Oligocene^2,14^. Most of the sediments from Central Vietnam were supplied to the Mekong River Basin, and the amount of sediment delivered to the Yinggehai Basin was limited^11,57^. Jonell et al. (2017) suggested that Central Vietnam had a slow regional bedrock exhumation rate throughout the Cenozoic based on detrital apatite fission track and ^10^Be data in the modern sediments, and 17–21% of the total sediments now preserved in the Yinggehai Basin may have been provided by CVS^41^. Therefore, Central Vietnam was a minor source of the sediments in the Yinggehai Basin, and it had little influence on the characteristics of the sediments in the Qiongdongnan Basin^2,14,23,41^. Different sources with positive or negative Eu anomaly characteristics can be identified by REE analysis. Furthermore, the sediments carried by the Red River exhibit positive Eu anomalies, while those derived from granitic and sedimentary parent rocks on Hainan Island exhibit negative Eu anomalies^24^. The provenance evolution from the Oligocene to the Pliocene can be explored based on the our geochemical data and REE data from other studies in the Yinggehai and Qiongdongnan basins.

**Figure 10:**
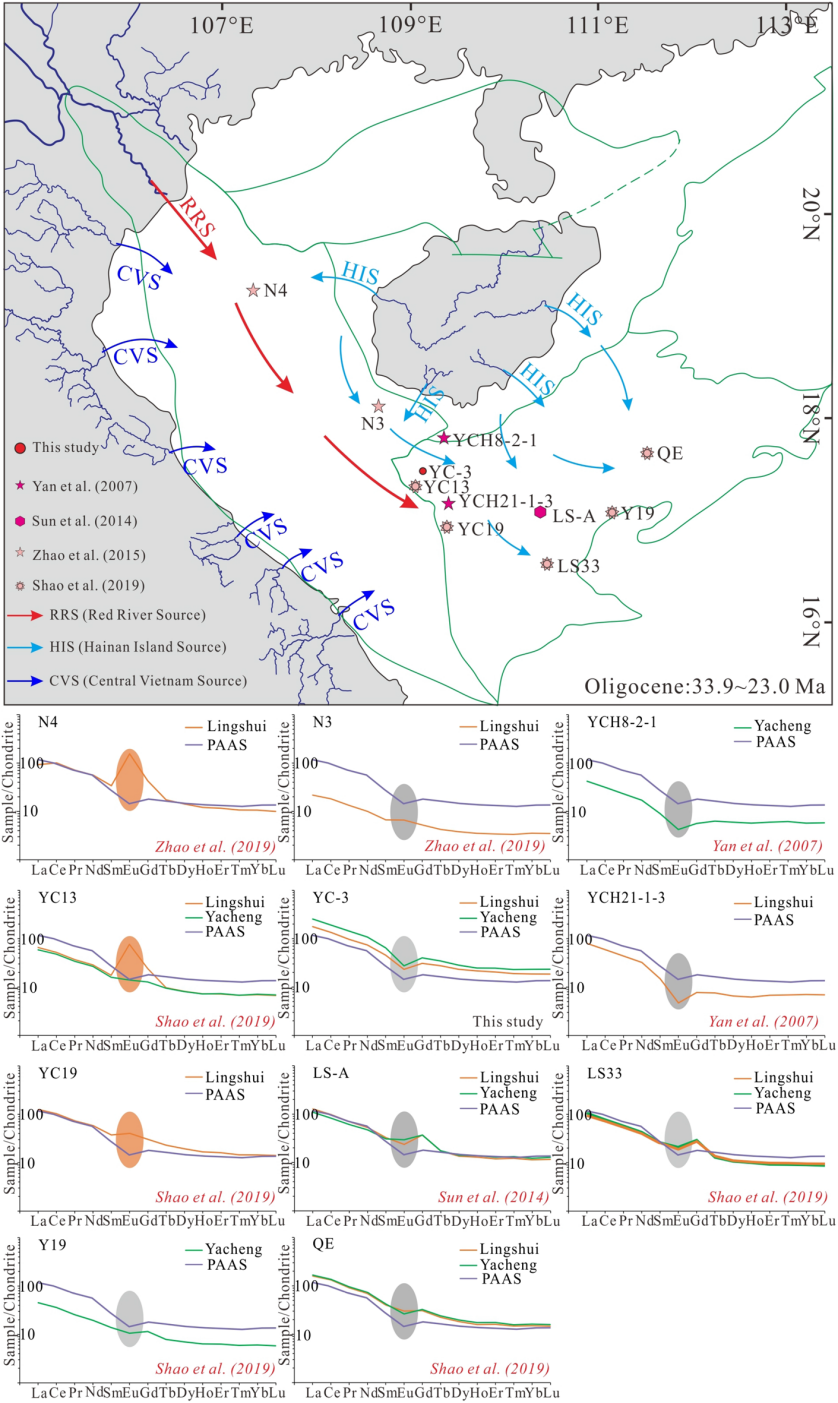
Schematic source-to-sink evolution model and chondrite^50^ normalized REE patterns of Oligocene samples from the Yinggehai and Qiongdongnan basins. Eu-anomaly values are highlighted on each plot with gray (negative) or orange (positive) shades. The square part of the Yinggehai and Qiongdongnan basins in the upper map to display the provenance distribution. Intervals in each REE pattern plot indicate the Formation analyzed. PAAS—Post-Archean Australian shale.

Based on the REE patterns of the potential sources and the sediments deposited during the Oligocene (33.9 ~ 23.0 Ma; Fig. 10), the samples from wells N4 in the Central Depression of Yinggehai Basin and YC13 and YC19 in the junction area of the two basins exhibit positive Eu anomalies, and are similar to the RRS, which indicates that sediments deposited in the Central Depression of the Yinggehai Basin and the junction area of the two basins may have the contribution from the RRS. While the other samples from wells N3, YCH8-2–1, YCH21-1–3, YC-3, LS-A, LS33, Y19, and QE have negative Eu anomalies and are similar to the HIS. It is suggested that the sediments deposited in the Yingdong Slope of the Yinggehai Basin and the Qiongdongnan Basin during the Oligocene were mainly delivered from the HIS (Fig. 10). Overall, the RRS provided a great of the sediments for the Central Depression of Yinggehai Basin and the western part of Qiongdongnan Basin, while the sediments from the HIS were mainly deposited on the Yingdong Slope of Yinggehai Basin and the Qiongdongnan Basin, and the junction area of the two basins had a mixed sediments source consisting of the RRS and the HIS.

**Figure 11:**
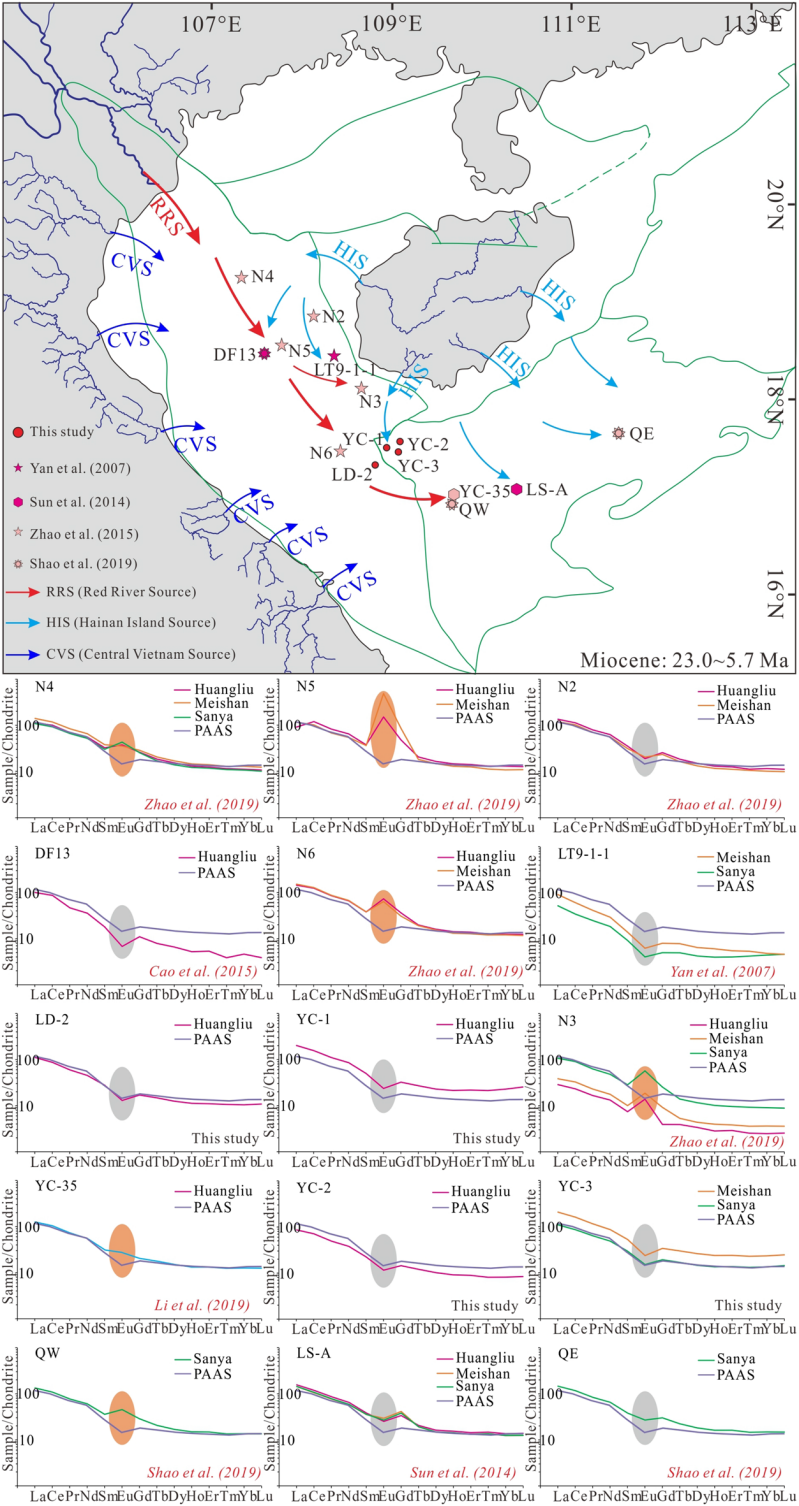
Schematic source-to-sink evolution model and chondrite^50^ normalized REEpatterns of Miocene samples from the Yinggehai and Qiongdongnan basins.

During the Miocene (23.0 ~ 5.7 Ma; Fig. 11), the sediments from wells N4, N5 and N6 exhibit positive Eu anomalies, while those from wells DF13 and LD-2 exhibit negative Eu anomalies in the Central Depression of Yinggehai Basin, which indicates that both the RRS and HIS provided sediments to the Central Depression of Yinggehai Basin (Fig. 11). Except for well N3, the Miocene sediments from other wells in the Yingdong Slope exhibit negative Eu anomalies, suggesting that the sediments in this area are not solely derived from the HIS, and another possible source is the RRS (Fig. 11). The sediments from wells QW and YC-35 exhibit positive Eu anomalies, similar to the positive Eu anomalies of RRS, while the sediments from the other wells in Qiongdongnan Basin with negative Eu anomalies, and are similar to the HIS (Fig. 11). The RRS only provided sediments for the western part of Qiongdongnan Basin. Consequently, the RRS and HIS may have had a combined effect on the composition of sediments in the Central Depression and Yingdong Slope of the Yinggehai Basin and the junction area of the two basins.

**Figure 12:**
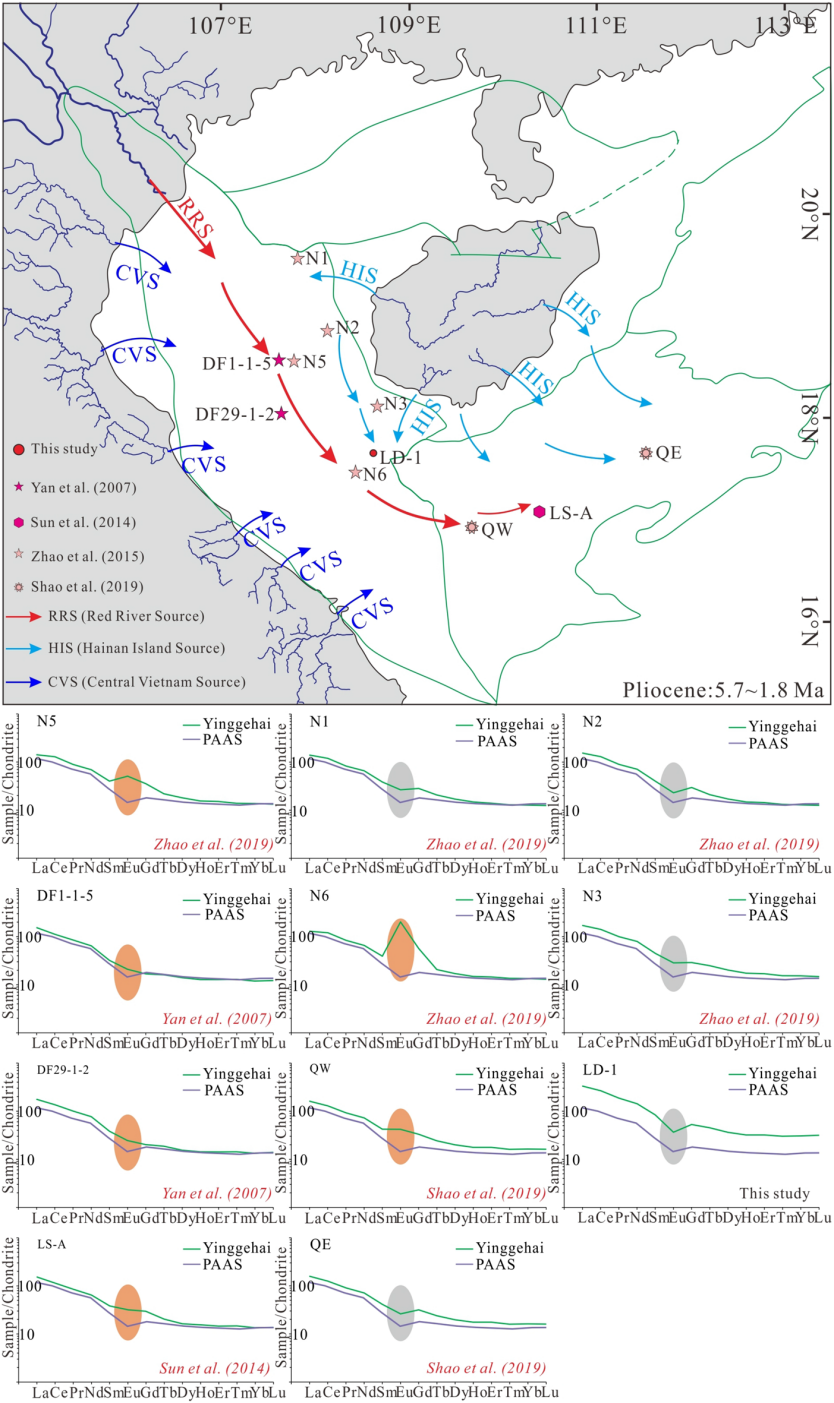
Schematic source-to-sink evolution model and chondrite^50^ normalized REEpatterns of Pliocene samples from the Yinggehai and Qiongdongnan basins.

During the Pliocene (5.7 ~ 1.8 Ma; Fig. 12), except for samples from well LD-1, all samples (wells N5, DF1-1–5, DF29-1–2, and N6) in the Central Depression of the Yinggehai Basin exhibit positive Eu anomalies. From north to south in the Yingdong Slope, the sediments from wells N1, N2, and N3 all exhibit negative Eu anomalies, suggesting that the sediments deposited on the Yingdong Slope during the Pliocene were derived from the HIS (Fig. 12). In the western sags in the Qiongdongnan Basin, the sediments from wells QW and LS-A exhibit positive Eu anomalies. However, well QE was characterized by negative Eu anomalies, which indicates a different source for the sediments in the eastern and western parts of Qiongdongnan Basin. Most of the sediments deposited in the eastern part of Qiongdongnan Basin during Pliocene were derived from the HIS (Fig. 12). In general, the sediments deposited in the Central Depression of Yinggehai Basin and the western part of Qiongdongnan Basin were derived from the RRS, while the sediments deposited on the Yingdong Slope of Yinggehai Basin, northern and eastern parts of Qiongdongnan Basin during Pliocene were primarily derived from the HIS.

From the Oligocene to the Pliocene (33.9 ~ 1.8 Ma), the RRS provided sediments to the Central Depression of the Yinggehai Basin and western part of the Qiongdongnan Basin. Meanwhile, most of the sediments preserved in the Yingdong Slope of the Yinggehai Basin and the Qiongdongnan Basin were derived from the HIS. During the Miocene (23.0 ~ 5.7 Ma), the RRS and HIS were also one of the sedimentary sources of the Yingdong Slope and Central Depression of the Yinggehai Basin, respectively. Last but not the least, the junction area of the two basins had a mixed source consisting of the RRS and HIS during the Cenozoic (33.9 ~ 1.8 Ma).

## Conclusions

The major and trace elemental characteristics of Cenozoic sediment samples from the junction area of the two basins reveal that they were derived from continental island arc to passive continental margin settings.

The REE distribution patterns of sediments from the junction area of the two basins are nearly parallel and with large negative Eu anomalies, indicating they are derived from Hainan Island with exposed igneous and ancient sedimentary rocks.

During the late Cenozoic (33.9 ~ 1.8 Ma), the RRS provided sediments to the Central Depression of the Yinggehai Basin and the western part of Qiongdongnan Basin, the HIS provided sediments to the Yingdong Slope of the Yinggehai Basin and Qiongdongnan Basin; However, the RRS and HIS were also one of the sediment sources of the Yingdong Slope and Central Depression of the Yinggehai Basin during the Miocene (23.0 ~ 5.7 Ma), respectively; Furthermore, the junction area of the two basins had a mixed source consisting of the RRS and HIS during the Cenozoic.

## Supplementary Information

Supplementary Information.

## Data Availability

All data generated or analyzed during this study are available in Supplementary Table 1.
